# A populational review of the amyloid precursor protein gene mutations relevant to alzheimer’s disease

**DOI:** 10.1192/j.eurpsy.2021.1902

**Published:** 2021-08-13

**Authors:** I.M. Balmus, A. Ciobica, L.D. Gorgan

**Affiliations:** 1 Department Of Interdisciplinary Research In Science, Alexandru Ioan Cuza University of Iasi, Iasi, Romania; 2 Center Of Biomedical Research, Romanian Academy, Iasi, Romania; 3 Department Of Biology, Faculty Of Biology, Alexandru Ioan Cuza University of Iasi, Iasi, Romania; 4 Department Of Biology, Academy of Romanian Scientists, Bucharest, Romania

**Keywords:** Alzheimer’s disease, amyloid precursor protein, Populational Genetics

## Abstract

**Introduction:**

The genetic component of Alzheimer’s disease was previously studied and more than sixty amyloid precursor protein (APP) gene mutations were identified. However, the populational aspects of this component were scarcely discussed despite that many of the reports mentioned the demographic ancestry of the carriers or probands.

**Objectives:**

In this short study, we aimed to review the APP gene mutations relevant to Alzheimer’s disease from a Populational Genetics point of view by evaluating the current literature for the demographic description of the carriers or families in which the mutations were identified.

**Methods:**

In this regard, multiple genetic studies on the APP gene mutations relevant to Alzheimer’s disease were reviewed and the incidence of the mutations was analyzed considering the ancestry of the patients.

**Results:**

We found many possible scenarios regarding the incidence of the APP gene mutations in Alzheimer’s disease patients and general population. On the one hand, we could identify several mutations which were present in more than one population (eg. V615M, V717I, V717L) and on the other hand, some mutations could be observed in certain populations (eg. E693delta, the Osaka mutation, which was until now observed in Japanese patients, while E693G was found in a Swedish family). One particular case is that of the isolated populations (eg. the Icelandic population in which an APP mutation protecting against Alzheimer’s disease is more frequent in the general population as compared to the patients).

**Conclusions:**

We were able to identify several mutations which were characteristic to many populations, but also some population-specific features regarding the APP genotypes.

**Disclosure:**

No significant relationships.

